# Endosymbiont or host: who drove mitochondrial and plastid evolution?

**DOI:** 10.1186/1745-6150-6-12

**Published:** 2011-02-19

**Authors:** Jeferson Gross, Debashish Bhattacharya

**Affiliations:** 1 Department of Ecology, Evolution and Natural Resources, and Institute of Marine and Coastal Sciences, Rutgers, The State University of New Jersey, Foran Hall 102, New Brunswick, NJ 08901, USA

## Abstract

The recognition that mitochondria and plastids are derived from alphaproteobacterial and cyanobacterial endosymbionts, respectively, was one of the greatest advances in modern evolutionary biology. Researchers have yet however to provide detailed cell biological descriptions of how these once free-living prokaryotes were transformed into intracellular organelles. A key area of study in this realm is elucidating the evolution of the molecular machines that control organelle protein topogenesis. Alcock et al. (Science 2010, 327 [5966]:649-650) suggest that evolutionary innovations that established the mitochondrial protein sorting system were driven by the alphaproteobacterial endosymbiont (an "insiders' perspective"). In contrast, here we argue that evolution of mitochondrial and plastid topogenesis may better be understood as an outcome of selective pressures acting on host cell chromosomes (the "outsiders' view").

**Reviewers:**

This manuscript was reviewed by Gáspár Jékely, Martijn Huynen, and Purificación López-García.

## Introduction

### From inside or outside, how to reconstruct organellogenesis?

Modern-day plastids and mitochondria are fully integrated into the regulatory networks encoded by the nuclear genome [1]. Yet these compartments descend from free-living Gram-negative bacteria that were taken up by "host" cells as endosymbionts and transformed into intracellular organelles [1-5]. During this process of organellogenesis, a nucleus-to-organelle flow of biogenetic information was established through the progressive ability to direct nuclear-encoded proteins synthesized in the cytosol to specific subcellular locations within the endosymbionts [1,2,5]. Such a regulated protein topogenesis within the nascent organelles was made possible by the evolution of specialized molecular machines that comprise the ancestors of the modern-day mitochondrial and plastid protein sorting apparatuses. In mitochondria, these are the translocons of the outer and inner mitochondrial membrane (Tom and Tim23, respectively), the Tim22 insertase, the sorting and assembly machinery (Sam), and the small Tim chaperones [1,5,6]. In plastids, protein sorting is under control of the translocon of the outer and inner membranes of the chloroplast (Toc and Tic, respectively) [1,3].

In an attempt to advance our understanding of organellogenesis and invigorate discussion on this topic we recently proposed a new model (termed the "outsiders' hypothesis") [1]. This model posits that genetic integration and the establishment of protein sorting systems in both the plastid and mitochondria occurred in a step-wise evolutionary trajectory, with the host guiding molecular components first to the outer membrane (OM) of the endosymbiont, and then to the intermembrane space (IMS), inner membrane (IM), and finally to the organelle interior. Such an outside-to-inside evolutionary trajectory seems to us to be inherently appealing because one cannot easily imagine a selective pressure that would result in the establishment in the nucleus of a gene encoding a protein that operated in the IM of the organelle (e.g., a solute carrier) if host-encoded proteins could not first efficiently cross the OM of the endosymbiont. In addition, our model recognized that by being held captive inside the host cell the plastid and mitochondrial forerunners were subject to the typical genomic "meltdown" universally observed among obligatory prokaryotic endosymbionts (see below) [7-9]. Selection over the maintenance of the consortium led the host to progressively assume control over decaying organelle functions [1]. Therefore the outsiders' view favors the idea that evolutionary novelties leading to the establishment of the organelle were mainly selected on the host chromosome. The outsiders' hypothesis contrasts with traditional models to explain the evolution of mitochondria and plastids that are united by the view that organelle protein sorting systems originated to target nuclear-encoded proteins into the endosymbiont interior (e.g., in the prokaryotic cytosol [10-14] or in its IM [15]). These insiders' models usually entail that molecular components were established in the endosymbiont chromosome to drive protein import into the nascent organelle [5,10,12-18] (although some descriptions emphasize the role of genetic innovations occurring on the host chromosome; e.g., ref. [15]). This insiders' view is adopted by Alcock and colleagues [5], who suggest that components still encoded on the putatively minimally reduced alphaproteobacterial endosymbiont genome were "tinkered" with by evolution to import nuclear-encoded proteins [5,19,20]. Thereby it is assumed that the alphaproteobacterium had an important, if not preponderant, role in its conversion into an organelle. In this opinion piece we discuss these endosymbiont- or host-driven views of organelle evolution in light of current data from molecular phylogenetics and genome analyses of eukaryote organelles and endosymbionts to assess which may provide a better platform to understand the complex process of organellogenesis.

## Discussion

### Endosymbionts: evolutionary novelties or genome erosion?

Prokaryote endosymbionts are found in association with diverse hosts in the eukaryote tree of life [7,21,22]. Given that plastids and mitochondria descend from endosymbionts, it is logical to pose the following questions: what can we learn from extant prokaryotes that exist in an intracellular association with eukaryotes, and, is there evidence for selection resulting in genetic tinkering with their genomes to create new molecular machines? It is now well established from a plethora of comparative genome studies that the universal trend among endosymbiotic bacteria is the steady accumulation of deleterious mutations, high rates of nucleotide substitutions (reflected by a high AT-content), and progressive genome shrinkage [7,8,21]. These aspects of genome erosion are explained by a Muller's ratchet process that affects endosymbionts because of their small population size and isolation within host cells where they lose access to external sources of DNA to repair newly arisen deleterious mutations [9]. Accordingly, the strength of genetic drift is proportional to the degree of host-endosymbiont interdependency, with facultative endosymbionts being less affected than obligatory endosymbionts. The latter typically have genomes with high AT-contents and show dramatic size reduction (< 1 Mbp) [7,21]. Such elevated genetic drift is characterized by lower efficacy for selection even with regard to essential genes [7]. Nonetheless selection exists, however it is acting primarily to counterbalance genetic drift. One excellent example is the widespread tendency of purifying selection over maintenance of protein coding regions to withstand the ratchet of high nucleotide substitution rates [7,8]. Similarly chaperones tend to be constitutively over-expressed in endosymbionts to buffer the overall thermal instability of the proteome due to high rates of amino acid substitution [7,23]. Only in rare instances are newly arisen adaptive traits (though not new molecular machines) observed in the endosymbiont genome. An example is the duplication of biosynthetic genes presumably to boost the output of metabolites essential for the endosymbiotic association [24]. Taken together the existing data point to a clear trajectory for endosymbionts: genome decay. This milieu may therefore not provide an ideal test bed for the evolution of novel molecular machines *via *tinkering.

### The cyanelle of *Paulinella chromatophora*: a new plastid tinkered from inside or forged by genetic drift?

The filose amoeba *Paulinella chromatophora *possesses a plastid-like photosynthetic compartment (the cyanelle or chromatophore) that was recently (ca. 60 Mya) derived from a *Synechococcus*-like cyanobacterial endosymbiont [25,26]. Although protein import into the putative chloroplast-type compartment has not yet been demonstrated, several observations indicate the cyanelle of *P. chromatophora *has already reached the status of a *bona fide *organelle [27-29]. These are: synchronized division of the cyanelle with the host cell cycle, the paucity of cyanelle-encoded metabolite transporters, and, more important, the documented evidence of endosymbiotic gene transfer (EGT) (e.g., genes encoding the subunits of the photosystem I, *psa*E, *psa*I, *psa*K, and high-light inducible proteins). This putative "retelling the tale" of plastid evolution in the Plantae (Archaeplastida) provides an excellent case study to determine whether genetic tinkering played a role in the early stages of organellogenesis. Consistent with its recent endosymbiotic past, the genome of the cyanelle bears the footprints of genetic drift that affects genomes of intracellular prokaryotes; i.e., accelerated nucleotide substitution rates, high AT-content, dramatic genome reduction (about 1/3 of the size of free-living *Synechococcus *strains), gene inactivations, widespread gene deletions, and purifying selection over protein coding regions [25,27,29,30]. With the exception of a fusion of the *ftn2 *gene with its neighboring open reading frame (PCC0126), thus far there is no bioinformatic evidence for the evolution of novel organelle-derived functions in this genome *via *for example, gene duplications or horizontal gene transfer [27,30]. In fact, accompanying the loss of 2/3 of the cyanobacterium-derived genome, many key genes needed to sustain a free-living life style have been jettisoned, leaving reduced material to tinker with. Therefore *P. chromatophora *once again points to the prominent role that genetic drift plays in shaping the evolution of an emerging organelle genome.

### Mitochondrial and plastid organellogenesis: an insiders' or outsiders' tale?

**Figure 1:**
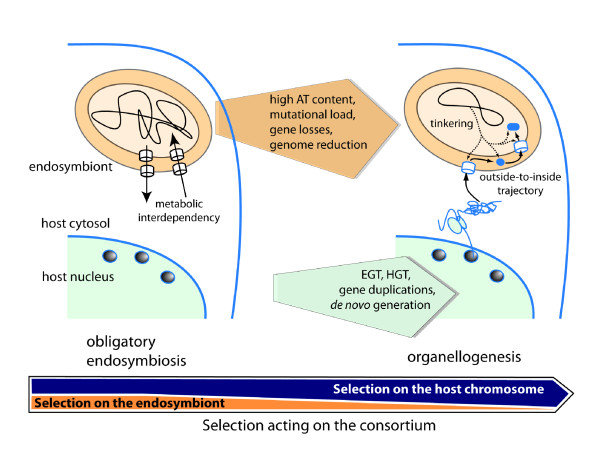
**The outsiders' model for the conversion of a prokaryotic endosymbiont into an organelle**. Schematic figure showing the process of organellogenesis, whereby a eukaryotic cell (shown in cross-section) harbors an obligatory Gram-negative endosymbiont (left) that is converted over time into an organelle (right). Obligatory endosymbiosis (right) implies mutualistic benefits that usually include metabolic interdependency between the host and its endosymbiont. At this point, selection is strong over both the host and the prokaryote to maintain or improve the benefits of the consortia (see arrow down). Genetic drift however leads to progressive genome degeneracy of the endosymbiont. This is marked by an increase in AT content, mutational load, losses of genes, and pronounced genome reduction. Strong selection for the maintenance and increase in fitness of the consortia progressively favors selection on the host chromosome. We suggest that in some cases this eventually may lead to the process of organellogenesis (right), which presumably initially includes the establishment in the host chromosome of molecular components to drive the biogenesis of OM proteins of the endosymbiont. Organellogenesis then progresses in an outside-to-inside trajectory of establishment in the endosymbiontic compartments of host-encoded molecular factors (blue) synthesized in the cytosol. This process is driven by EGT, HGT, gene duplication, subfunctionalization, and *de novo *generation of genes. Organellogenesis may eventually include the establishment of proteins that are tinkered from inside the organelle. The arrow down represents a trend of selection acting on the consortia, which tentatively can be described as a combination of selection acting on the endosymbiont (which tend to decrease over time) and of selection on the host chromosome (which tend to predominate during organellogenesis). The arrows indicate that the process unfolds over time (from left to right) and not to depict an actual relative time scale of the process of organellogenesis. It is possible that the period of obligatory endosymbiosis could have been relatively short compared to the time frame necessary for full evolution of an organelle such as the plastids and mitochondria. Note that the figure suggests a model useful to describe the evolution of any eukaryotic organelle derived from a prokaryotic endosymbiont. However it is not clear whether the host of the mitochondrial forerunner was a *bona fide *eukaryote or an Archaea in an ongoing process of evolving a nuclear compartment [1,4].

Given that plastids and mitochondria descend from captive prokaryotes, there is no *a priori *reason to believe their evolutionary trajectory would differ markedly from modern-day endosymbionts and from the cyanelle of *P. chromatophora*. In fact, evidence exists that Muller's ratchet is counterbalanced in plastid genomes by polyploidy and gene conversion [31] and still influences the evolution of mitochondrial genomes [32]. Therefore it is reasonable to assume that the precursors of mitochondria and plastids were similarly affected by genome degeneracy and loss of fitness due to being held captive in the host's cytosol (Figure 1). This led to an evolutionary trajectory marked by genome reduction, suggesting that endosymbiont-derived genomes have never been an ideal arena for the evolution of new molecular components through tinkering. The outsiders' perspective however acknowledges that evolution of a prokaryotic-derived organelle follows the dynamics of the endosymbiotic process (Figure 1). Intracellular resident prokaryotes often establish an obligatory syntrophic association with their host cells [7,21,22,33]. It is conceivable that the alphaproteobacterial precursor of mitochondria was exchanging metabolites derived from its aerobic metabolism (e.g., tricarboxylic acids) with a putative archaeal host [4]. Similarly, the cyanobacterial forerunner of plastids probably extruded photosynthates used in the cytosol of the host [34]. Therefore maintenance of the fitness of the cyanobacterial and alphaproteobacterial endosymbionts was likely crucial for survival of the corresponding hosts [1]. What would result if endosymbiont fitness was progressively compromised by genetic drift? We suggest this resulted in strong selective pressure on the host chromosomes to evolve molecular components that assumed control over decaying prokaryotic biogenesis, setting organellogenesis in motion (Figure 1) [1]. Porins are beta-barrel proteins in the OM of Gram-negative prokaryotes that support the first layer of metabolic flow regulation of cells with the external milieu [35,36]. Given the importance of porins and the fact that host-encoded factors only had access to the OM of the endosymbiont, we previously proposed that mitochondrial and plastid evolution might have been initiated by the host exerting control over the topogenesis of OM proteins (i.e., beta-barrel proteins) of the captive prokaryotes [1]. This ensured an immediate regulation over the metabolic flow across the endosymbiont's OM. We propose that this involved the establishment of OM pores (e.g., Tom40 in the emergent mitochondrion and Toc75 in the plastid) and the co-option of Omp85 homologs (Sam50 in mitochondria and putatively one of the Toc75 homologs in plastids) from the alphaproteobacterial and cyanobacterial endosymbionts, respectively, to drive the catalytic assembly of beta-barrel proteins. Organellogenesis then progressed by an outside-to-inside establishment of molecular components that led to the evolution of modern organelle protein sorting systems. How this happened involved a substantial amount of genetic tinkering (that arguably could have occurred on the host genome, see below) as well as the establishment of key new genes.

### Tim23: endosymbiont or host origin?

The insiders' hypothesis makes a straightforward prediction: core molecular components involved in protein topogenesis are derived from the endosymbiont [5,19]. Important in this respect are the origins of Tim17, Tim23, and Tim22 that comprise a family of pore subunits in the IM of mitochondria [1,16]. Tim17 and Tim23 form the protein-conducting channel of the Tim23 translocon, whereas Tim22 is the pore of the insertase for mitochondrial carriers. The idea that Tim17, Tim23, and Tim22 originated *via *tinkering with the LivH family of bacterial amino acid transporters has been used to support the insiders' perspective for the evolution of mitochondria [5,10,15,16,19,37]. Similarly, a purported relationship of Tic20, a subunit of the protein translocon at the inner membrane of plastids, with LivH homologs has also been taken as an example of tinkering from "inside" during plastid evolution [17,18,38], and even during organellogenesis of the *P. chromatophora *cyanelle [39]. However BLAST searches against the NCBI protein database reveal that neither Tim17/23/22 nor Tic20 share any obvious sequence similarity with LivH homologs [1,3]. The original proposition in 1999 that LivH homologs were progenitors of Tim17, Tim23, and Tim22 relied on a partial alignment of 52 amino acid residues from 5 proteins and it was unclear whether this was explained by homology or potential convergence at the conserved sites (e.g., in the trans-membrane regions) [16]. The possibility that endosymbiont-encoded ancestral amino acid transporters were adapted to transport host proteins is clearly an attractive idea, however there is currently a lack of unambiguous evidence that LivH is the candidate protein [1,3]. An alternative interpretation is that the progenitor of Tim17, Tim23, and Tim22 might have been established (either by *de novo *generation or by HGT/EGT from a prokaryotic progenitor and since then underwent divergent evolution) in the chromosome of the alphaproteobacterial host, and Tic20 in algae and plants is probably unrelated to this family of proteins.

### Organelle protein topogenesis: tinkering from inside or outside?

Protein-coding genes that trace their origin to non-coding DNA (i.e., *de novo *provenance) are an important source of genetic novelty in prokaryotes and eukaryotes [40,41]. Apart from Tim17/23 and Tim22, many proteins that lack homologs in prokaryotes likely arose in eukaryotes to support organelle protein translocation (e.g., Tom20, Tom22, Mia40, Erv1, Tim54, Tim21, and the small Tims in mitochondria; Toc34, Tic110, Tic40 in plastids) [1,3]. These may have been generated *de novo *in the eukaryotic genome or derived from prokaryotic sources by HGT/EGT and since then diverged beyond recognition of the ancestral form. In fact, phylogenetic studies indicate that a large fraction of mitochondrial proteins originated after the acquisition of the alphaproteobacterial endosymbiont [42,43]. One excellent example of newly evolved proteins in the host chromosomes are provided by the family of IM mitochondrial carriers [44]. Similarly, the majority of solute permeases in the IM of plastids are derived from the host chromosome, in this case by another pathway: duplication of genes encoding existing vacuolar or plasma membrane transporters and the co-option of paralogs for plastid functions [45]. This suggests a major trend in organellogenesis whereby the host cell assumes control over the exchange of metabolites in the mitochondrial and plastid IM by reprogramming the permeome of the respective alphaproteobacterial and cyanobacterial endosymbionts using host-derived solute transporters [1]. In addition, phylogenetic studies reveal that a large proportion of both mitochondrial and plastid proteomes is composed of proteins derived from prokaryotes *via *HGT [42,43,46]. Because HGTs, *de novo *generation of ORFs, and gene duplications are rare in the genomes of obligatory endosymbionts [7], the chimeric nature of the mitochondrial and plastid proteome is better explained by the hypothesis that the arena for genetic tinkering was in the genome of the eukaryotic host and not that of the captured endosymbionts (Figure 1) [1].

In light of this perspective how should we interpret the fact that protein sorting components in both the mitochondrion (e.g., Sam50, Tim44, Oxa) and the plastid (e.g., Toc75, Tic20, Hsp93) are derived from the alphaproteobacterial and cyanobacterial endosymbionts, respectively [1,3,10,18]? As recognized by Jacob [47], the tinkering process involves duplication of the original gene followed by subfunctionalization of a paralog [41]. Gene duplications are a predicted intermediate step in the process of EGT, whereby a copy of the organelle gene is established in the nucleus prior to its loss from the endosymbiont genome [2,27,48]. If we assume selective pressures over the host chromosomes, it follows that these random duplications of organelle genes in host genomes provided the raw material for evolution of new subunits to drive organelle topogenesis. EGT demonstrates that genetic tinkering was an important aspect driving organellogenesis that can be interpreted as having occurred outside the organelle (i.e., in the host genome).

## Conclusion

### A consensus: tinkering inside in an outsiders' context

The central idea of the insiders' view that protein sorting in mitochondria was cobbled together from pre-existing components in the chromosome of the alphaproteobacterial endosymbiont [5] raises an interesting question: how can this process be described in a step-wise manner? For example, which would be the first endosymbiont-derived component to be recruited to import host-encoded proteins from the cytosol? A first approximation would likely lead to the same conclusion as the outsiders' hypothesis; i.e., the first protein component to be "tinkered" with probably was the OM channel Tom40 because protein sorting to inside the organelle requires that host proteins could first cross an open gate in the OM. Because host-encoded proteins need progressively to gain access to the endosymbiont, it is reasonable to suggest that the proposed endosymbiont-guided tinkering process unfolded in an outside-to-inside trajectory. Nonetheless theses ideas bring to light yet another basic contradiction. If such strong selective pressures over the endosymbiont chromosome efficiently converted endogenous factors into import machines, then why are these factors no longer encoded in any of the mitochondrial genomes sequenced to date? The observation that both the plastid and mitochondrial protein-sorting components are universally encoded in the nucleus [1-3,10,15,37] implies that selection to establish control of organelle topogenesis in the host chromosome was decisive during organellogenesis. In conclusion, even a strict endosymbiont-dominated insiders' view needs to incorporate the premises of the outsider's hypothesis (i.e., the outside-to-inside trajectory and host-control over organellogenesis) in order to interpret the current state of organelle topogenesis.

We suggest that by assuming an outsiders' perspective it is possible to gain insights into the contribution of "tinkering" inside the endosymbiont during organellogenesis (Figure 1). For example, it is conceivable that the pores Tom40 and Toc75, in the OM of mitochondria and plastids, respectively, could have initially evolved in the chromosome of the endosymbiont and facilitated the docking and permeation of the nuclear-encoded copies of Tom40 and Toc75 once these components had been established *via *EGT. The progenitors of the mitochondrial matrix processing peptidase (MPP) and the plastid stromal processing peptidase could also provide examples of adaptations that occurred inside the endosymbionts in a later stage of organellogenesis to cleave pre-sequences of import substrates inserted into the IM from outside. However the more complex process of duplication and subfunctionalization of the alphaproteobacterial peptidase that produced the modern MPP heterodimeric form [49] most likely occurred in the nuclear genome of the host. Under this perspective organellogenesis, like any complex evolutionary process, reflects the sum of different relative contributions made by the participating genomes (Figure 1).

In conclusion, this opinion piece focuses on what we suggest to be a useful paradigm for understanding organelle evolution. We believe that the main merit of our model is to overcome several concerns we have about the insiders' views and to recognize the importance of stepwise, gradual evolution. The outsiders' view, however like all hypotheses needs testing. An important step towards this goal will be achieved by proteomic and comparative genomics studies of endosymbiotic bacteria and the nuclear genomes of their hosts. Of special interest are the new candidates for organellogenesis such as the cyanobacterial-type resident of *Rhopalodia gibba *diatom [50], the psyllid endosymbiont *Carsonella ruddii *[51], and, in particular, the nuclear genome of *P. chromatophora *and the proteome of its plastid-like cyanelle [25,26,29]. Given the breadth of genome projects that are currently underway and the rise of single cell genomics [21,52], we anticipate that the next few years will provide unprecedented opportunities to significantly improve and broaden our understanding of the host-endosymbiont divide.

## Competing interests

The authors declare that they have no competing interests.

## Authors' contributions

JG and DB conceived the hypothesis and wrote the manuscript. Both authors read and approved the final manuscript version.

## Reviewer's comments

### Reviewer's report 1

*Dr. Gáspár Jékely, Max Planck Institute for Developmental Biology, Tübingen, Germany*.

In this short paper Gross and Bhattacharya elaborate on their previous publications about the origin of organellar targeting during early eukaryote evolution. Although there is not much new information in this paper, I support publication, because the authors address specific criticism in a recent Opinion article appeared in Science about their scenario (ref. [5]). This is a good opportunity to elaborate these arguments, and I would like to contribute to it with some comments.

#### Author's response

*We appreciate the referee's comments and we opportunity to further illustrate key ideas of our model*.

I think that the authors have to be more specific about the use of the terms 'outsiders' and 'insiders' hypothesis'. The problem is that they confound three very different questions. The first question is whether the components of the organellar targeting machineries have an origin from the symbiont's or the host's genome.

#### Author's response

*We think this first question is not a confusion raised by our analysis, because the origin of the molecular components should ultimately be determined empirically using phylogenetic analysis. From the perspective of the outsiders' hypothesis, molecular components can derive from either the host, the endosymbiont, or from unrelated taxa by HGT. Important to our argument is selection acting on the host to establish these components. From the perspective of some insiders' views (e.g., ref. *[5]*) the core molecular components must derive from the endosymbiont. This is why confusion was generated regarding the origin of the Tim17/Tim23/Tim22 family. To help clarify this point, here we bring into question the proposed origin of this family from endosymbiotic (prokaryotic) amino acid transporters*.

The second is whether during the conversion of the symbiont into an organelle the first steps entailed changes in OM components or the IM/matrix components of the symbiont.

#### Authors' response

*In this regard, we note a substantial difference between our model and traditional views of organelle evolution. The label "outsider" refers to the fundamental idea that conversion of the endosymbiont into an organelle was progressively driven from outside-to-inside the endosymbiont. It started in the OM of the captive endosymbiont, and then to the inner membrane space (IMS), IM, and finally to the organelle interior. Therefore it is not only the question of where organellogenesis started, but actually how the process of organellogenesis was organized in a directional step-wise manner. This idea is in sharp contrast with all models suggested so far because they collectively argue that protein sorting systems in both plastid and mitochondria were somehow adapted to import proteins to inside the organelle (e.g., the IM or the organelle interior) *[5,10,12-18].

The third question is whether the mutational changes occurred in the host or mitochondrial genome.

#### Author's response

*"Outsider" also refers to the idea that most of the evolutionary novelties leading to the establishment of the organelle arose "outside" the endosymbiont: i.e., in the genome of the host. We agree that many models also include this idea (e.g., ref. *[15]*, discussed below), they however either do not describe the process in question or tend to resort to the notion of tinkering on the inside. Our description of the endosymbiont-to-organelle conversion as a coherent step-wise genetic integration of prokaryotic functions into the host chromosome makes a strong case for a host-guided process and thereby provides a robust description of organellogenenesis in a way not seen in previous models*.

There are many combinations of the possible answers to these three questions. Various combinations have been proposed in various models in the literature. These distinctions have to be clarified in the text. Once it is done, it is clear that the differences between the authors' model and other models are not so clear-cut. Therefore the authors cannot lump all other models together as "insiders' models" and then argue against all of them at the same time.

#### Author's response

*We respect the referee's opinion and we have now attempted to make a clearer distinction in the introduction about the differences between the outsiders' versus the insiders' perspectives regarding what the reviewer refers to as the "second and third questions" (although we don't feel it is within the scope of our manuscript to review one-by-one the publications on the topic of evolution of protein sorting in organelles). We do agree with the referee that nuances between outsiders' and insiders' positions exist and we acknowledge the possibility of limited tinkering from inside the organelle (now discussed). However we feel that traditional models collectively fail to recognize a directional outside-to-inside trajectory for the establishment of molecular factors in the endosymbiont and that the genetic integration of the organelle functions in the host chromosomes follows this pattern. We feel that the scope of the outsiders' view and the possibility of a detailed description of organellogenesis that it offers contrasts sufficiently with previous models of organelle evolution that it is fair to distinguish them as we have done. Once some key differences (discussed below) are understood we hope the contrast between our model and the traditional views will become clearer*.

*(i) To our knowledge, with the notable exception of Cavalier-Smith's model for the origin of the protein sorting components in mitochondria (criticized below), the insiders' models fail to provide an account of the order in which the molecular components forming the protein import machines were established. In other words, they do not incorporate a process-like description. Most of the insiders' models rely on an "adaptive" argument that if molecular machines were already present in the endosymbiont they could "somehow" be easily adapted to operate protein import inside the organelle (i.e., the tinkering argument) *[5,10,12-14,18,20]*. Thereby many insiders' models claim that evolution could be gradual, however they do not provide an account of the steps involved in such gradual tinkering*.

*(iI) The outside-to-inside perspective is supported by current experimental data on mitochondrial protein import (see ref. *[1]*and refs. therein). Some examples are worth to be reinforced. One of them is the well established interplay between the biogenesis of Sam50 and Tom40, which is interpreted by the outsiders' view as an ancestral and essential system that evolved at the onset of mitochondrial organellogenesis. The fact that the small Tim chaperones in the IMS are essential to support the functions of the OM and IM biogenesis suggests this system served as a key intermediate development during evolution of host-control over the endosymbiont membranes. There is a fundamental connection between protein sorting in the IM and protein translocation into the matrix that manifests gradual outside-to-inside evolution. This is represented by the common phylogenetic origin and biogenetic interdependency of Tim22 and Tim23 machines. In this context, the fact that Tim23/Tim17 complex is an insertase and only acts as an translocase by addition of the PAM module at the mitochondrial matrix illustrates the concept of a molecular machine gradually evolving from outside-to-inside. An interesting fact is that Tim23 serves as an insertase for proteins containing a single transmembrane domain (STMD), which are abundant in the complexes of the respiratory chain. In addition, in many taxa the subunits of the matrix processing peptidase that cleaves off pre-sequences of import substrates are components of the respiratory chain complex III. These correlations highlighted above suggest that protein sorting in mitochondria evolved to support host-control of organellar oxidative phosphorylation. Finally, the fact that the hosts of both the alphaproteobacterial and cyanobacterial endosymbionts "reprogrammed" the prokaryotic permeome with host-derived solute transporters may be tentatively explained by at least two outsiders' interpretations. One is the obvious host-control over the metabolic flow across the organelle membranes. The second might be a topological constraint to insert from outside molecular components derived from the endosymbionts, since the original prokaryotic transporters were assembled from inside*.

*(iii) In contrast to previous suggestions that speculate independently about the evolution of mitochondria or plastids our hypothesis attempts to understand the evolution of any endosymbiotic-derived organelle of prokaryotic origin. We suggest clear parallels in how the evolution of the mitochondria and plastids proceeded. In addition, the outsiders' hypothesis elaborates on the notion implied from modern comparative genomic studies with endosymbiontic bacteria that genetic drift eroding the prokaryotic genome might have been an important factor underling the endosymbiont-to-organelle conversion*.

*(iv) The ultimate root of the insiders' view is the signal hypothesis that initially intended to describe how a protein is targeted to the interior of the endoplasmic reticulum *[53]*. Thereby it was assumed that the inherent function of a protein sorting system is to direct proteins to the lumen of an organelle. Traditional models for the evolution of protein topogenesis in mitochondria and plastid adhered to this idea and aimed at providing an insiders' description of organelle function and evolution. The outsiders' hypothesis can bring a fresh perspective to understanding the essential organization of a protein sorting system. For example, it is by applying a gradual outside-to-inside description of the evolution of protein sorting components that one can rationalize why precursor of proteins destined to the OM, IMS, and the mitochondrial carriers do not have N-terminal extensions (pre-sequences). We suggest that pre-sequences appeared in a later stage to facilitate the insertion of STMD-containing proteins into the IM by the Tim23/Tim17 insertase, and only a posteriori did they acquire the "meaning" of a signal. As a consequence we suggest that pre-sequences are actually signals for insertion into the IM and that the genuine signal for protein import into the matrix of mitochondria that emerged during evolution was the absence of an alpha-helical transmembrane domain following the pre-sequence. Such a hypothesis that topogenic signals emerged from functional traits of the imported proteins is to our knowledge novel and merits discussion*.

To give an example, the model by Cavalier-Smith [ref. [15]] agrees in many points with the authors' model. He also proposed for example that Tom20, Tom22, Sam37 and mitochondrial carriers originated and evolved in the host genome.

#### Author's response

*The referee raises and important example to be contrasted with our outsiders' hypothesis because Cavalier-Smith's model seems to be the only additional attempt so far made to provide a detailed description for the evolution of a protein sorting system in an organelle. Although Cavalier-Smith's model has many merits, in our opinion it has fundamental problems that stem from assuming insiders' premises. For example, while considering that Tom20, Tom22, Sam37 evolved in the genome of the host, Cavalier-Smith also adheres to the idea that Tim17/23 and Tim22 evolved from endosymbiotic LivH homologs. He also proposes that the small Tim chaperones are of prokaryotic origin, and that Tom40 is derived from the usher-type secretion component of Gram-negative bacteria, even if there is no rigorous phylogenetic support for such suggestions. In addition he argues that the YidC homolog of the alphaproteobacterium was inserting mitochondrial carriers arriving from outside the cell. To our knowledge, such an inverted topology of YidC homologs has not yet been observed in prokaryotes or mitochondria. These examples (and below) illustrate how insiders' views can confuse phylogenetic and cell biological phenomena while attempting to explain how the endosymbiont was importing host proteins*.

The major difference between Cavalier-Smith' model and the model by Gross and Bhattacharya regards the first steps in the process (carriers insertion versus Tom40 and SAM50 self-insertion into the OM). I can see the merits of the first solution (immediate selective advantage of ATP/ADP or other exchanges), but I don't see the advantage to the host of exchanging the OM beta-barrel proteins (this was also not discussed in the authors' Nat Rev Genet paper ref. [1]). Could it have been advantageous for the control of transport, or organelle division? I am just guessing, but this point could be elaborated in this manuscript. I would argue that the first steps had to involve a mechanism to control or tap the evolving organelle, and I don't see the solution from a pure outsider (OM targeting first) perspective.

#### Author's response

*In principle the OM of Gram-negative bacteria is not permeable to external proteins. It is not clear in Cavalier-Smith's model how a carrier protein translated into the cytosol of the host could have gained access to the IM of the alphaproteobacterium. It is suggested that the Tom40 pore was permeable to the carriers. However evolution has no foresight, therefore the establishment of Tom40 must have been selected for other purpose that preceded the insertion of carriers in the IM. In addition it is assumed that chaperones in the periplasm of eubacterial origin guided the carriers to the IM where they were inserted by LivH homolog (the supposed Tim22 progenitor). Periplasmic membrane chaperones are directional, in a sense that they take substrates present by the Sec translocase at the IM and escort them to the OM *[54]*. This function may be accomplished by crossing the peptidoglycan layer. Finally, as we discussed in the text, the origin of Tim22 from LivH has not yet been proven*.

*Besides the idea that initially the insertion of an ATP/ADP translocator was not feasible, the outsiders' hypothesis relies on the notion that a metabolic cycling between the host and the alphaproteobacterial endosymbiont already existed before organellogenesis. This is a characteristic of obligatory endosymbiotic relationships of modern prokaryotes residing within host cells (*Figure 1) [7,33]. *We assume that the survival of the metabolic association, whatever it was, was crucial for both the host and the endosymbiont. As discussed in the text, as a result of a Muller's ratchet the endosymbiont's genome tend to decay. This is as well expected to compromise the fitness of the host. In many instances observed in modern host-endosymbiont associations the solution to such a problem is the acquisition of a new endosymbiont *[7]*. In rare cases, the outcome might be the transformation of the endosymbiont into an organelle *[21,51]*. We suggest this happened with the alphaproteobacterial and cyanobacterial forerunners of mitochondria and plastids, respectively *[1]*. If this was the case, where then molecular components of the host are expected to access the endosymbiont functions in the first place? Porins in the OM of Gram-negative bacteria are often regarded as unselective sieves for metabolites. However the expression of porins in the OM of eubacteria is exquisitely regulated and mutations affecting porin permeability often affect metabolite flow rates *[35]*. In addition the mitochondrial VDAC porin is as well subjected to fine regulation *[36]*. The fact that the OM is the first layer of metabolite exchange control of Gram-negative bacteria and that it is the only layer topologically accessible to host cytosolic components, suggests that establishment of a host-encoded system to control beta-barrel biogenesis in the alphaproteobacterial OM is the first predictable target for selection to improve the fitness of the host-endosymbiont association. In the new manuscript we elaborate the ideas described above with the support of a figure*.

In the conclusions the authors state that: "what were the selective advantages conferred to the endosymbiont that led it to relinquish autonomy by importing host-proteins to manipulate its biochemistry?" This is not a valid question in this context. Once the symbiont had established an obligatory presence in the host, selection acted at the level of the consortium (host+symbiont) as a unit. It is therefore conceivable that certain mutations in the organellar genome were fixed, even if such mutations would have harmed a free-living cell or a facultative symbiont. If mutations in the organellar genome for example changed the properties of the OM pores and made it easier for the host to insert carriers and thereby increase the fitness of the consortium, such mutations could have spread. Nevertheless I acknowledge the other valid arguments on why the symbiont's genome was likely not contributing with major innovations during the process.

#### Author's response

*The selection over the consortia is certainly the dominant perspective. This idea can also be described as a central tenet of the outsiders' hypothesis if we however understand that the concept of selection over the consortia should be broken and analyzed into its components; i.e., a host-level selection and the evolutionary dynamics of the endosymbiont (see *Figure 1*). It is clear nowadays from genomic studies with modern endosymbiotic bacteria that intracellular prokaryotes are under a neutral process of evolution (i.e., genetic drift). This led, we suggest, to an increased selection over the host to assume control of the prokaryotic functions. In other words, selection over the host chromosome was the major component of a selection to maintain or increase the fitness of the consortia (*Figure 1*). Despite this trend, the referee clearly makes an important point, and now we acknowledge that instances of tinkering from inside could be possible if understood under the view of progressive control exerted by the host over endosymbiont functions. This idea is now discussed in the last section of the revised manuscript. With regard to the question quoted by the referee, we agree it is indeed inappropriate and has been omitted from the manuscript*.

In the section "Organellar protein topogenesis" the authors mention *de novo *provenance from non-coding DNA as a potential source of new genes. A cautionary note here: it is more likely that these proteins evolved from preexisting proteins and diverged beyond recognition, rather than evolved *de novo*. It is in general very difficult to evolve folded proteins from scratch.

#### Author's response

*We mention now the possibility that certain proteins lacking prokaryotic similarities could nonetheless be of prokaryotic origin, but have diverged beyond the point of recognition of the ancestral form. However cases of new proteins emerging from non-coding DNA do exist in the recent literature *[41].

### Reviewer's report 2

*Prof. Martijn Huynen, Nijmegen Center for Molecular Life Sciences & Center for Molecular and Biomolecular Informatics, Nijmegen, Netherlands*.

The manuscript by Jeferson Gross and Debashish Bhattacharya concerns the discussion about whether endosymbiosis of mitochondria and plastids was driven by the host or by the endosymbiont. The hope is that if we can trace the evolutionary events that transformed the free-living bacteria into organelles we can understand the original rationale for that transformation for both partners in the endosymbiosis events. What separates organelles from (intracellular) bacteria is the import and functioning in the organelle of nuclear encoded proteins, and thus the discussion focuses on the machinery that imports those proteins and its origin. Gross and Bhattacharya have recently proposed the outsiders' hypothesis, which argues that this machinery originated with nuclear encoded proteins (albeit of potentially endosymbiotic origin), and that these proteins first provided access to the outer membrane, then the intermembrane space, then the inner membrane and then the inside of the organelle. This view is in contrast with one in which the proteins encoded on the endosymbiont would have evolved to import nuclear encoded proteins, before their genes would have been transferred to the nucleus. In the latter view, at least in the paper by Alcock et al (ref. [5]), the first membrane that is decorated with new proteins during the endosymbiosis is the inner membrane, to allow the transport of metabolites that presumably would pass through existing channels in the outer membrane.

This is all a rather academic discussion as we cannot go back to the past. Furthermore most of the theoretical arguments have already been spelled out and need not to be restated by me. Finally I will not comment on the "logic" of either scenario, as evolutionary Biology to me often only makes sense in hindsight.

What is left for me to comment on are the new arguments that the authors bring to the table. I do agree that the homology of LivH with Tim23 is rather contentious, although the authors might have used a more thorough analysis than pairwise sequence similarity analysis using Blast. But, not only using pairwise sequence comparisons, but also profile-profile analysis (this referees' analysis) fail to detect any significant homology, even when the search is restricted to the putatively homologous region originally identified by Rassow et al (ref. [16]). This putative homology is often quoted, and I for one never realized that there was so little supporting evidence.

#### Author's response

*We thank the referee for this important contribution*.

The authors' examination of the *Paulinella chromatophora *cyanelle as an example of what happens if the tape is played twice sounds convincing, but should include a statement that, despite all the indirect evidence in that direction, there is no documented case of protein import into *P. chromatophora *yet.

#### Author's response

*We note now the absence of direct evidence for protein import into the cyanelle of Paulinella chromatophora*.

Finally, the authors' argument that among genes encoded on organellar genomes there are no protein sorting components is convincing at least in the sense as it shows that there is no direct evidence for a "tinkering inside" hypothesis with respect to protein transport.

Although there is a clearly observable trend of genome decay in mitochondrial genomes, I do not think that that is necessarily all there is. There are examples of nucleus to mitochondrial genome transfer, and there are many ORFs in mitochondrial genomes, e.g. in ciliates, of which the origin and potential function are unknown.

#### Author's response

*The cases of nucleus-to-mitochondrion gene transfer that the referee alluded to are likely to concern higher plants whose mitochondrial genomes have the exceptional ability to take up DNA by HGT *[55]*. The interesting case of ORFs of unknown function in ciliates might as well represent a feature exclusive to a specific taxonomic group in which the organelle is experiencing high rates of sequence divergence. In Tetrahymena species 20 out of 44 encoded ORFs are of uncertain provenance *[56]*. However in other non-ciliate mitochondria the number of ORFs with undetermined function and origin is much reduced. For example, the 97 genes encoded in the Reclinomonas americana organelle represent the largest set of mitochondrial ORFs known thus far. Only 3/97 represent ORFs of unclear function and provenance *[57]*. This suggests that many ORFs in the mitochondria of ciliates and other species may be a result of ancestral proteins that have diverged beyond recognition. An example is provided by Ymf66 in Tetrahymena thermophila that has marginal similarity to the mitochondrial Fo subunit restricted to very few functionally important amino acids and the overall polytopic transmembrane structure *[58]*. Alternatively ORFs of unknown origin may represent instances of HGT or tinkering inside the organelle exclusive to the ciliate clade*.

Could the authors comment on the *Buchnera Daphnia *symbiosis (or others with obligatory intracellular endosymbionts). There is evidence there for the sharing of amino acids. Probably the import/export machinery of amino acids requires less tinkering of proteins than the import/export of proteins, and therewith could reflect "tinkering inside". I am not suggesting that the mitochondrial carrier family is of endosymbiotic origin, but also a protein like ATM1 that is required for cytosolic FeS assembly, that exports a metabolite from the mitochondrion, and that is clearly of endosymbiotic origin, argues to consider the transport of metabolites separate from the transport of proteins.

#### Author's response

*A hallmark of obligatory associations involving eukaryotic host cells and endosymbionts is the cycling of metabolites between the symbiotic partners *[7,33]*. The amino acid interdependence cited by the referee is broadly observed in associations involving aphid hosts (sap-feeding insects) and Buchnera species (gammaproteobacteria). In many instances the endosymbiont provides essential amino acids absent in the host diet. In addition genome studies have uncovered that the host-endosymbiont amino acid interdependence tend to be based in complementary metabolic pathways *[7,33]*. For example, whole or partial biosynthetic pathways that are not encoded in the genome of the prokaryote are provided by the host or potentially by a second endosymbiont. Knowledge about the molecular components in the membranes of the endosymbionts involved in the transport of metabolites is still scarce. It is likely that in the majority of cases the set of transporters encoded on the endosymbiont genome might suffice to regulate cross-flow of metabolites. Alternatively, as suggested by the referee, it is possible that limited modifications of transporters encoded on the endosymbiont genome might modify a pre-existing molecular component to sustain essential exchange of metabolites. Intriguingly, the paucity of transporters encoded in some endosymbionts (e.g., Sulcia muelleri *[33]*and Carsonella ruddii *[51]*) already suggests ongoing processes of organellogenesis in which permeases controlling host-endosymbiont metabolite exchange might presumably be encoded on the host genome *[51]*. These examples highlight the importance of modern-day intracellular associations of prokaryotes within eukaryotic host as excellent model systems to investigate to which degree tinkering from inside and/or host-level selection contributes to the evolution of the obligatory consortia*.

*Nuclear-encoded prokaryote-derived components that exert functions in modern-day organelles, such as the family of ABC transporters to which ATM1 belongs *[59]*, are abundant in mitochondria and plastids. These might represent instances of EGT/HGT potentially followed (or not) by gene duplications and subfunctionalization and therefore probably reflect tinkering on the host chromosomes, as discussed in the text*.

In line with this I am a bit puzzled about the strong distinction that is made between the "tinkering from inside" and "tinkering from outside". It comes down to where (in which genome) exactly the mutations occurred that allowed the transport of cytoplasmic proteins into the mitochondrion (as stated by the authors). If the endosymbiont and the host became completely dependent upon each other before the development of a new protein import machinery, e.g. by export and import of metabolites as has been proposed for Buchnera, we are already dealing with one species, with very limited genetic heterogeneity among the endosymbionts and thus also very little possibility of adaptive evolution at that level. Thus an argument regarding the selective advantages of releasing autonomy (page 10) would no longer be valid.

#### Author's response

*We provide now a longer discussion and a figure to highlight the nature of selective pressures that may have led to the transformation of the aphaproteobacterial and cyanobacterial endosymbionts into the mitochondria and plastids, respectively. In our opinion it is clear that selection acted on the consortium. However this general phenomenon, we believe, is dominated by host-level selection that is crucial to understand organellogenesis (*Figure 1*)*.

### Reviewer's report 2, revised manuscript

The only point I would like to clarify is with respect to the reaction on my comment about nuclear-to-mitochondrial genome gene transfer. The latter has not only been observed in plant mitochondria, but also in some corals for MutS (Pont-Kingdon et al. J Mol Evol. 1998 Apr;46(4):419-31), or in some fungal mitochondria that contain an RNA polymerase containing plasmid, that at least in one case has been integrated in the mitochdrial genome (Formighieri EF et al. Mycol Res. 2008 Oct;112(Pt 10):1136-52).

#### Author's response

*We thank the referee for this comment. We agree that evolutionary novelties can exist in modern-day organelles and also occurred in the past. However it seems to us that the examples cited here represent sporadic phenomena uncovered in specific taxa and do not represent a general tendency in modern-day mitochondria. Important to our consideration of organelle evolution is the widely applicable idea that nuclear control over mitochondrial and plastid functions was a key step in the evolution of these organelles. This idea entails that most genetic novelties that play a role in organelle evolution occurred in the nuclear genome*.

### Reviewer's report 3

*Dr. Purificación López-García, Universite Paris-Sud, Paris, France*.

This manuscript is a reply to the commentary by Alcock et al. (2010, Science 327:649·650) to a previous opinion article by J. Gross and Bhattacharya (2009, Nature Reviews Genetics 10:495·505), where these authors already exposed the hypothesis that mitochondrial and plastid evolution was driven by the host, which imposed the protein-sorting machinery in a step-wise process starting from the outer membrane towards the interior of the organelle (the "outsider's" view).

#### Authors' response

*We wish to make a brief comment about use of the phrase by this reviewer that the host "imposed" protein sorting components to the endosymbiont; this may sound like a "parasitic" interaction. Given the level of genome degeneracy that affects obligatory endosymbionts, a description that more properly illustrates our model is that the host "rescued" endosymbiont topogenesis by establishing a protein sorting machinery in the nascent organelle*.

Alcock et al. presented an opposed view (the "insider's" view), implying that mitochondrial and plastid protein translocation machineries would have been mostly derived from existing endosymbiont bacterial transporters. This manuscript is just a prolongation of that discussion, though it has the merit of raising the divergences between the two views in a direct way and highlighting some key unsolved issues. I have two general comments.

The major problem to test those models lies in the difficulties to trace the origin of the genes encoding the different components of the organelles' protein translocation systems. Gross and Bhattacharya are right in pointing out the problem of establishing a clear homology between some Tim and Tic components and the LivH family of bacterial amino acid transporters, since trans-membrane domains are often affected by convergence. However, I do not see why "In the absence of clear support for a prokaryotic origin, the most likely explanation is that Tim 17/23 and Tim22 were derived from the host of the mitochondrial forerunner...". For that, homology with host genes that predated the mitochondrial/plastid symbiosis should be established. This might be done in principle for plastid evolution, but not for mitochondria (unless primary amitochondriate eukaryotes are found some day). Actually, it could very well be that bacterial genes encoding membrane transporters and carriers were transferred by EGT to the host nucleus and evolved rapidly while a backup copy was still retained in the organelle. The nuclear-encoded proteins might have retained their specificity to interact with the former bacterial membrane while acquiring novel properties selected by the new needs of the consortium. In a way, the outside-to-inside view, which appears logical when thinking in terms of gene and genome evolution (it is indeed unlikely that gene tinkering occurred in the endosymbiont's genome) and host-endosymbiont interaction, might have been subsequent (or consequent) to an inside-to-outside process of EGT. Of course, this is hypothetical, and protein translocation machineries might have evolved from completely new genes (though, in the case of mitochondria, to what extent the evolution of new genes was not impacted directly or indirectly by the endosymbiosis?), but it is not possible to exclude that hypothesis with the data at hand.

#### Author's response

*We agree and discuss now in the text that absence of evidence for phylogenetic provenance does not prove that a gene was generated de novo. Proteins often strongly diverge from their ancestors, however the evolution of specific proteins in the host genome (whether generated de novo or diverging from EGT/HGT sources) is a trend that parallels organellogenesis and underlies a selection on the host chromosome. This is implied by the fact that several, if not hundreds, of new subunits from the respiratory complexes, ribosomes, and other multi-protein machines are functional in modern-day mitochondria, but are absent from the prokaryotic counterparts*.

*EGT should not generally be described as a form of inside-to-outside evolution. More likely, genes are established in the nucleus as a result of selective forces in the host genome, whereas genes are lost from the endosymbiont as a consequence of reduced selection (genetic drift). However selection on the host chromosome suggests that EGT represents a form of outside-to-inside evolution even if the organelle is the source of the nuclear gene copy. In addition, it should be noted that genes established in the chromosome of the host to maintain organelle functions represent, in a broader sense, the result of selection to maintain or improve the fitness of the consortia, as discussed in the text and depicted in *Figure 1.

My second comments relates to the propensity in the manuscript to separate host and endosymbiont's "interests" in an advanced state of symbiogenesis. For instance, for Gross and Bhattacharya "we come to the key question implied by the insiders' view that remains to be answered: what were the selective advantages conferred to the endosymbiont that led it to relinquish autonomy by importing host proteins to manipulate its biochemistry?". The question is not well formulated because as soon as an obligatory symbiosis establishes, which implies EGT of one or several essential genes to the host with the subsequent loss in the endosymbiont, selection begins to act at the consortium level. Likely, it was much more efficient in terms of the consortium energetic to reduce the bacterial genome to near extinction (or complete extinction, e.g. most mitochondria-derived hydrogenosomes). The question could only be asked for facultative symbionts, but facultative symbionts do keep, by essence, their autonomy; otherwise, they could not make a living on their own.

#### Author's response

*The quoted phrase was in fact a remark in the final section of our manuscript about some implications of Alcock et al. insiders' perspective which, by overemphasizing the active role of the endosymbiont, appears to imply such a "division of interests" *[5]*. We agree that the comment does not fit in a proper discussion of organellogenesis. In addition we provide now a longer discussion and the *Figure 1*emphasizing that the broader picture of organellogenesis is a selection over the evolution of the consortia, which entailed genetic novelties mostly occurring at the host chromosome-level*.
